# Distillation-free Scaling of Large State-Space Models for Images and Videos

**DOI:** 10.1007/s11263-026-02824-0

**Published:** 2026-04-17

**Authors:** Hamid Suleman, Syed Talal Wasim, Muzammal Naseer, Juergen Gall

**Affiliations:** 1 University of Bonn, Bonn, Germany; 2 Lamarr Institute for Machine Learning and Artificial Intelligence, Dortmund, Germany; 3 Department of Computer Science, Khalifa University, Abu Dhabi, United Arab Emirates

**Keywords:** Action Recognition, Mamba, Robustness

## Abstract

State-space models (SSMs), exemplified by S4, have introduced a novel context modeling method by integrating state-space techniques into deep learning. Despite their effectiveness, SSMs struggle with global context modeling due to data-independent matrices. The Mamba model addresses this with data-dependent variants enabled by the S6 selective-scan algorithm, enhancing context modeling, especially for long sequences. However, Mamba-based architectures face significant parameter scalability challenges, limiting their utility in vision applications. This paper tackles the scalability issue of large SSMs for image classification and action recognition without relying on additional techniques like knowledge distillation. We analyze the distinct characteristics of Mamba-based and Attention-based models, proposing a Mamba-Attention interleaved architecture that enhances scalability, robustness, and performance. We demonstrate that the stable and efficient interleaved architecture resolves the scalability issue of Mamba-based architectures and increases robustness to common corruption artifacts. Our thorough evaluation on the ImageNet-1K, Kinetics-400, and Something-Something-v2 benchmarks demonstrates that our approach improves the accuracy of state-of-the-art Mamba-based architectures by up to $+1.7$%.

## Introduction

Various networks have been proposed for both image and video recognition in recent years. These include convolutional neural networks (Krizhevsky et al., 2012; He et al., 2016; Carreira & Zisserman, 2017; Feichtenhofer et al., 2019), vision Transformers (Dosovitskiy et al., 2021; Arnab et al., 2021), and networks using focal modulation (Yang et al., 2022; Wasim et al., 2023). The Attention-based Transformer models have dominated both image and video recognition, either as pure Attention-based models (Liu et al., 2021, 2022; Arnab et al., 2021; Bertasius et al., 2021; Yan et al., 2022) or as hybrid models (Li et al., 2022b; Fan et al., 2021; Li et al., 2022a).

**Fig. 1:**
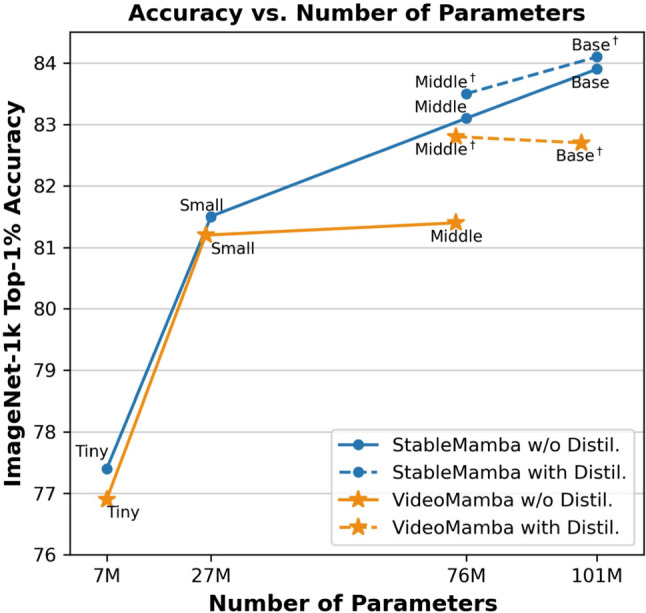
**Performance comparison with VideoMamba:** We compare the performance of our model with VideoMamba (Li et al., 2024), both with and without distillation, on IN1K (Deng et al., 2009)

Recently, State-Space Models (SSMs) such as S4 (Gu et al., 2022) have gained popularity as a new context modeling method. They recurrently model context and bring well-established techniques from state-space modeling to deep large models. However, S4 encountered a problem in terms of modeling global context due to the data-independent nature of the input, state-transition, and output matrices. To mitigate this issue, the Mamba (Gu & Dao, 2024) model introduced the S6 selective-scan algorithm, which uses data-dependent variants of the input and output matrices. This improves the context modeling capabilities, particularly on long sequences, and the approach has been adapted to image tasks (Zhu et al., 2024; Liu et al., 2024) and in the recent work VideoMamba (Li et al., 2024) to the video domain.

**Fig. 2:**
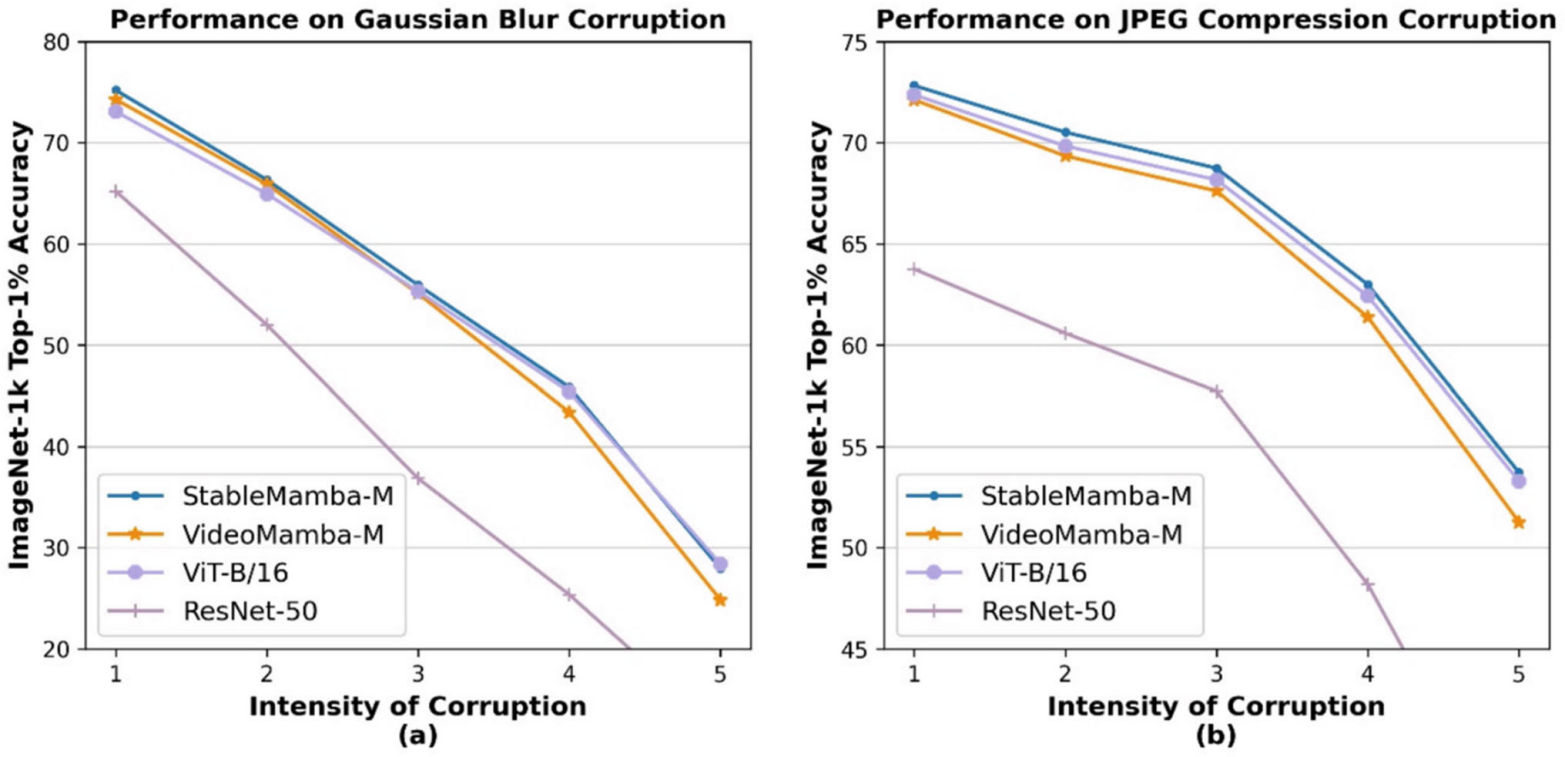
Performance comparison of different networks on Gaussian blur corruption. Performance comparison of different networks on JPEG compression corruption

In this work, we investigate the property of vision SSMs, where we focus on VideoMamba (Li et al., 2024) since it is the largest vision SSM architecture and the only one that can be applied to videos, and make two key observations. First, VideoMamba does not scale well with the number of parameters as plotted in Fig 1. While the accuracy substantially increases as the number of parameters is increased from 7M (tiny) to 25M (small) parameters, the accuracy only slightly increases if the parameters are increased further to 75M (middle) parameters. To mitigate this issue, Li et al. (2024) proposed to train first a small model and then use the small model as the teacher for training a larger model using distillation. While distillation improves the accuracy of the middle-sized model, it does not solve the underlying problem. Increasing the parameters further to 98M (base) parameters again does not improve the results.

**Fig. 3:**
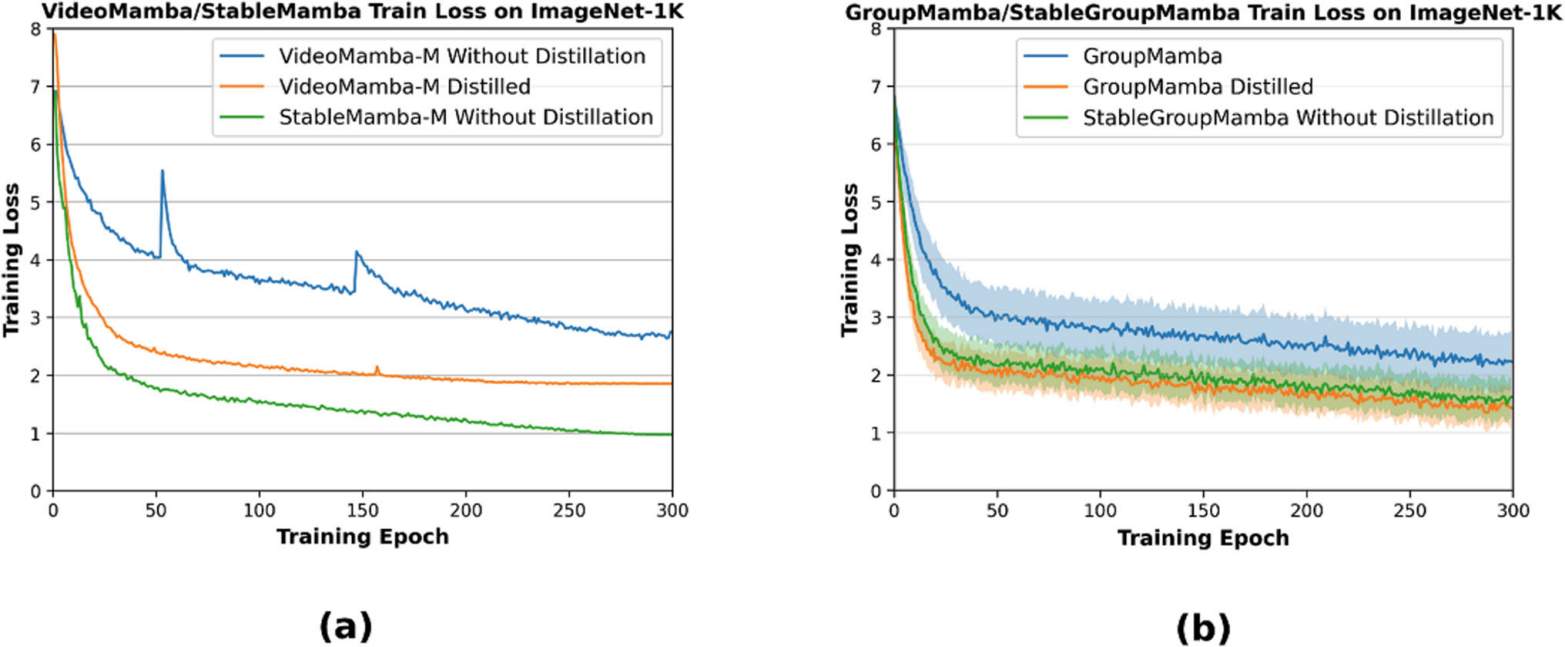
Loss curves obtained from training **a** VideoMamba with and without distillation against StableMamba. **b** GroupMamba-S trained with and without distillation as well as GroupMamba trained with our proposed method termed StableGroupMamba. Please see Section 5.1 for more details

**Fig. 4:**
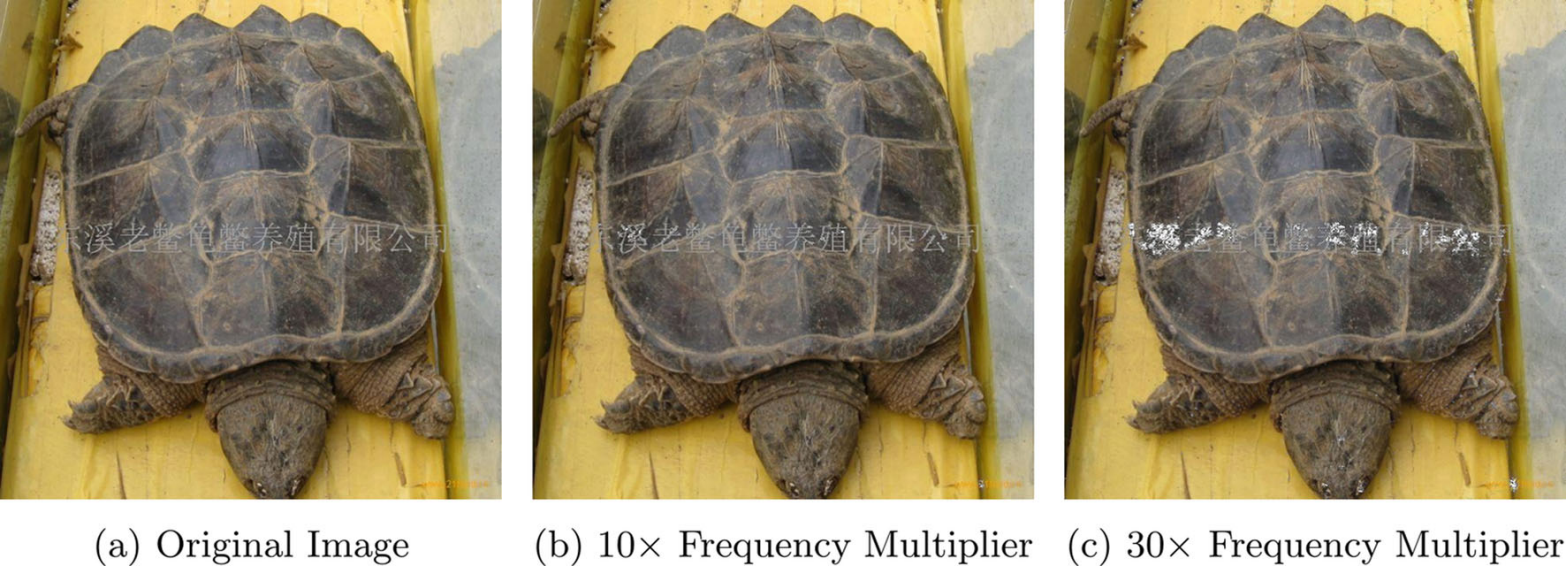
Visualization of high-frequency spectral amplification. Examples of the input perturbations used in the sensitivity analysis: **a** the original input image; **b** and **c** represent images where 20% of the highest frequency components are amplified by factors of 10 and 30, respectively. Although the fine-grained spectral noise shows only minor degradation of the image quality, VideoMamba is much more sensitive to such noise compared to ViT

The second observation is the higher sensitivity of the Mamba-based network to common corruptions and perturbations like image blur or JPEG compression in comparison to vision Transformers as shown in Fig 2. Both observations are major limitations for practical applications. We therefore propose a simple yet efficient Mamba-Attention interleaved architecture, termed StableMamba, that resolves both issues. It improves the robustness to common corruptions and perturbations during inference (Hendrycks & Dietterich, 2019) as shown in Fig 2 and mitigates the scalability issue without the need for cumbersome workarounds like distillation as shown in Fig 1. In summary, the main contributions of this paper are:

- We analyze the largest Mamba architecture for images and video and present a simple yet efficient Mamba-Attention interleaved architecture.
- We show that our approach resolves the scalability issue and increases the robustness to various common corruptions (Hendrycks & Dietterich, 2019).
- We report improved performance for comparable methods for image classification on ImageNet-1K (Deng et al., 2009) and for action recognition on Kinetics-400 (Kay et al., 2017) and Something-Something-v2 (Goyal et al., 2017).

**Fig. 5:**
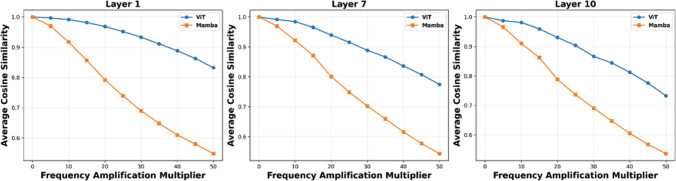
**Sensitivity analysis of internal representations to high-frequency spectral perturbations.** We plot the average cosine similarity between original token embeddings and those subject to varying levels of high-frequency amplification (the highest 20% of the spectrum). As the frequency amplification multiplier increases, VideoMamba exhibits a significantly sharper decline in similarity compared to ViT-B/16. This trend is consistent across early (a), middle (b), and late (c) layers, providing empirical evidence of Mamba’s high susceptibility to high frequencies

## Related Work

**Fig. 6:**
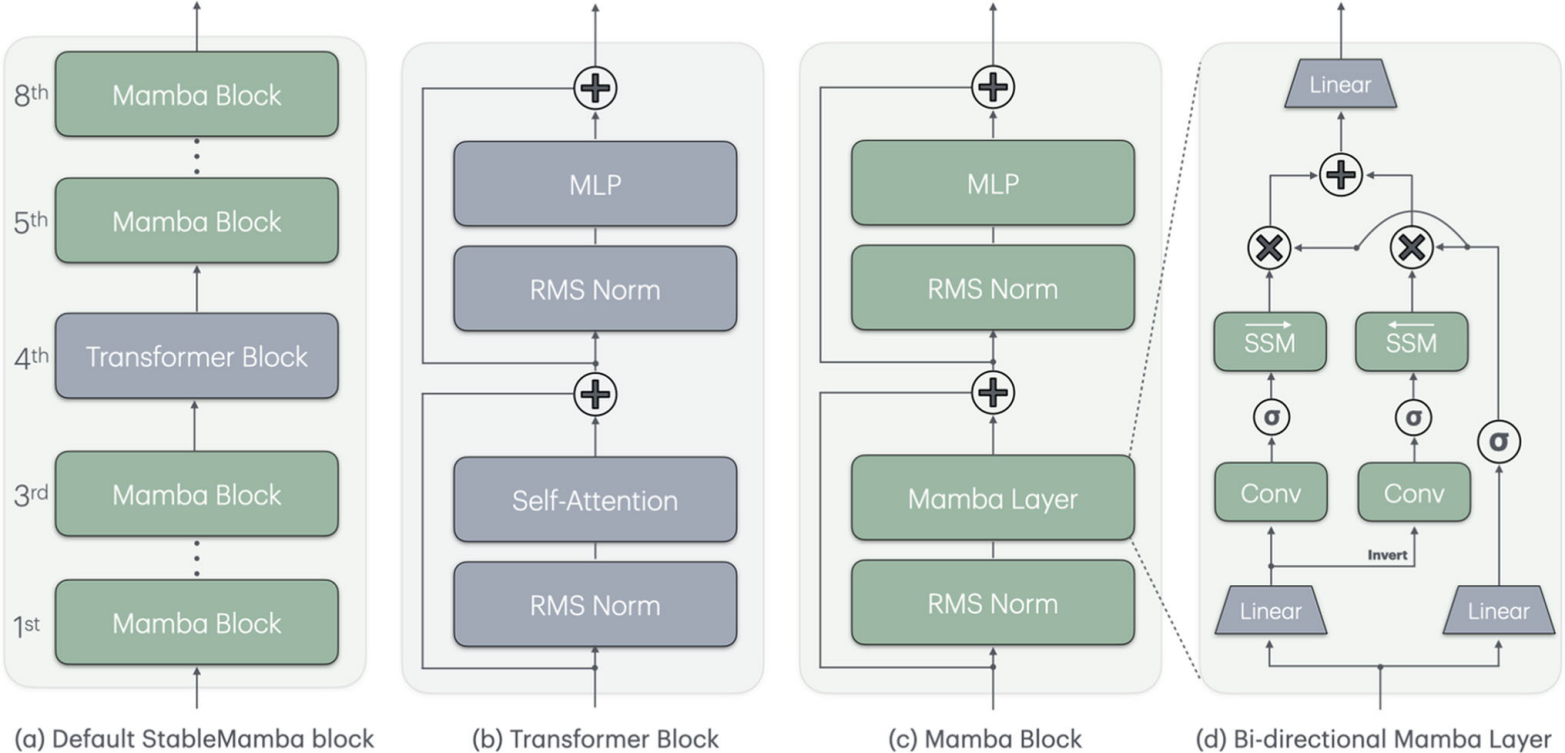
**a** The overall architecture of the StableMamba model. **b** Anatomy of the Transformer block. **c** Anatomy of the Mamba block. **d** Anatomy of bidirectional Mamba *layer*

**Image and Video Recognition:** In the last decade, Convolutional Neural Networks (CNNs) have been the primary choice for computer vision tasks. Starting with the introduction of AlexNet (Krizhevsky et al., 2012), the field has seen rapid advancements with notable architectures such as VGG (Simonyan & Zisserman, 2015), Inception (Szegedy et al., 2015), ResNet (He et al., 2016), MobileNet (Howard et al., 2017), and EfficientNet (Tan & Le, 2019) achieving improved performance on ImageNet (Deng et al., 2009). Recently, ConvNeXt variants (Liu et al., 2022a; Woo et al., 2023) and FocalNets (Yang et al., 2022) have updated traditional 2D ConvNets with modern design elements and training techniques, achieving performance comparable to state-of-the-art models. At the same time, the Vision Transformer (ViT) (Dosovitskiy et al., 2021), inspired by the Transformer (Vaswani et al., 2017) for natural language processing, and its variants such as DeiT (Touvron et al., 2021), Swin Transformer (Liu et al., 2021), and Swin Transformer V2 (Liu et al., 2022) have achieved very good results for image classification.

For Video Recognition, early methods were feature-based (Klaser et al., 2008; Laptev, 2003; Wang et al., 2013). Later, the success of 2D CNNs (Krizhevsky et al., 2012; Simonyan & Zisserman, 2015; He et al., 2016; Tan & Le, 2019) on ImageNet (Deng et al., 2009) lead to their application to video recognition (Karpathy et al., 2014; Ng et al., 2015; Simonyan & Zisserman, 2014). However, these methods lacked temporal modeling capabilities. The release of large-scale datasets such as Kinetics (Kay et al., 2017) prompted 3D CNN based methods (Carreira & Zisserman, 2017; Feichtenhofer et al., 2016; Tran et al., 2015). Since these were computationally expensive, various methods were proposed to mitigate the issue (Feichtenhofer, 2020; Sun et al., 2015; Szegedy et al., 2016; Tran et al., 2018; Xie et al., 2018; Li et al., 2020; Lin et al., 2019; Qiu et al., 2019; Feichtenhofer et al., 2019; Duan et al., 2020; Li et al., 2020; Wang et al., 2021). When the ViT (Dosovitskiy et al., 2021) architecture became popular in image recognition, it seamlessly made its way into the video domain. Initial methods used Self-Attention in combination with CNNs (Wang et al., 2018, 2020b; Kondratyuk et al., 2021) while later works (Liu et al., 2022b; Arnab et al., 2021; Bertasius et al., 2021; Yan et al., 2022; Zhang et al., 2021; Patrick et al., 2021; Fan et al., 2021; Li et al., 2022a; Patrick et al., 2021; Sharir et al., 2021) introduced pure Transformer based architectures. More recently, Video-FocalNets (Wasim et al., 2023) proposed a Focal Modulation (Yang et al., 2022) extension for videos, while Uniformer (Li et al., 2022b) proposed an efficient hybrid architecture for video recognition. Very recently, a key development in this area came with FlashAttention (Dao et al., 2022; Dao, 2023), which presents a hardware-aware implementation of the Attention algorithm that improves the efficiency of Attention-based models.

**State-Space Models:** Recently, State-Space Models (SSMs), such as the Structured State-Space Model S4 (Gu et al., 2022), have been presented as an alternative to Self-Attention (Vaswani et al., 2017) for efficient modeling of long sequences with linear complexity. Various variants building on the S4 architecture have also been proposed, including S5 (Smith et al., 2023), H3 (Fu et al., 2023), and GSS (Mehta et al., 2023). However, the original S4 (Gu et al., 2022) and its variants had a weakness compared to Self-Attention, mainly because they did not have any input dependencies. To mitigate this, Gu and Dao (2024) proposed the input-dependent state-space model Mamba alongside an efficient hardware-optimized parallel selective scan mechanism (S6). Various works have been proposed in computer vision applying Mamba to different downstream domains. Two variants were initially proposed for image classification: Vim (Zhu et al., 2024) and VMamba (Liu et al., 2024). Vim proposed an isotropic architecture with a bi-directional scanning variant of Mamba (Gu & Dao, 2024) for effectively scanning the image token sequence. In contrast, VMamba (Liu et al., 2024) proposed a hierarchical architecture with a four-directional scan across all four spatial dimensions. Subsequently, other variants such as LocalVMamba (Huang et al., 2024) had a Swin (Liu et al., 2021) style windowed scan while EfficientVMamba (Pei et al., 2025) proposed an atrous-selective scan to improve efficiency. The concurrent work GroupMamba (Shaker et al., 2025) proposed a parameter-efficient Modulated Group Mamba layer with channel grouping and distillation-based training. Furthermore, Mamba was also used in various applications in video understanding (Yang et al., 2024; Li et al., 2024; Chen et al., 2024), image segmentation (Liu et al., 2024; Ma et al., 2024; Ruan & Xiang, 2024; Gong et al., 2025), and various other tasks (Guo et al., 2024b; He et al., 2025; Wang et al., 2024; Guo et al., 2024a; Liang et al., 2024). SiMBA (Patro & Agneeswaran, 2024) uses the Fourier transform with non-linearities to model eigenvalues as negative real numbers in an attempt to improve the training. Similar methods have also been proposed for CNNs (Wang et al., 2020a) and Transformers (Xiao et al., 2021; Touvron et al., 2021). A complementary work to ours, VideoMamba (Li et al., 2024), proposes to use a distillation-based objective to stabilize the training of larger models. However, we show that a simple interleaving of Self-Attention layers within a Mamba-based model is enough to stabilize training for image and action recognition applications and improve robustness against high-frequency noise in the input. While prior works (Hatamizadeh & Kautz, 2025; Wang et al., 2024; Fei et al., 2024; Lenz et al., 2025) have explored hybrid Mamba–Transformer architectures, our contribution is distinct in both focus and scope. Specifically, we investigate the stability challenges that arise when scaling vision models, an aspect not addressed in these studies. For instance, JAMBA (Lenz et al., 2025) is an NLP-oriented work, while MambaVision (Hatamizadeh & Kautz, 2025), PoinTramba (Wang et al., 2024), MaTVLM (Li et al., 2025), and Dimba (Fei et al., 2024) do not analyze stability or evaluate model scaling in the image or video domain. PoinTramba has a specially different design adapted for point clouds where they insert entire encoders based on either Mamba or Transformer architectures unlike our interleaved design, among other differing components. Similarly, Dimba is designed for the diffusion process, where attention layers are substituted with Mamba layers to reduce the computational demand. JAMBA is a hybrid model for NLP, and MaTVLM is specifically made for VLM architectures while still employing distillation in design. Furthermore, MambaVision is a hierarchical model with convolutions as well as attention included in the design, unlike our isotropic design and strictly Mamba-Attention interleaved architecture. None of these works discusses the stability aspects of their designs. Our work is, to the best of our knowledge, the first to examine stability in large-scale vision models and to propose a hybrid design as a promising solution in this context.

**Table 1:** Hyperparameters for StableMamba

StableMamba Training Recipe			
T=Tiny, S=Small, M=Medium, B=Base, and L=Large			
Dataset	IN1K	K400	SSv2
Epochs	300	70(T), 50(S,M,B)	35(T), 30(S,M)
Batch size	128	32(T)/16(S,M,B)	32(T)/16(S,M)
Optimizer	AdamW	AdamW	AdamW
Optimizer momentum	$\beta _1=0.9,\beta _2=0.999$	$\beta _1=0.9,\beta _2=0.999$	$\beta _1=0.9,\beta _2=0.999$
Learning rate	5e-4	4e-4(T,S), 2e-4(M,B)	4e-4
Minimum learning rate	1e-5(T,S,M), 5e-6(B,L)	1e-6	1e-6
Scheduler	cosine	cosine	cosine
Weight decay	0.1(T), 0.05(S,M,B,L)	0.1(T), 0.05(S,M,B)	0.1(T), 0.05(S,M)
Warmup epochs	5 (T,S), 30(M), 20(B,L)	5	5
Trans. to Mamba blocks	1 : 7	1 : 7	1 : 7
Label smoothing	0.1	0.1	0.1
Drop path	0(T), 0.15(S), 0.5(M,B,L)	0.1(T), 0.35(S), 0.8(M,B)	0.1(T), 0.35(S), 0.8(M)
Repeated aug.	Yes(T), No(S,M,B,L)	2	2
Input size	$224^2$	$16\times 224^2$	$8\times 224^2$
Patch size	16	16	16
Rand. aug.	(7, 0.25)(T), (9, 0.5)(S,M,B,L)	(7, 0.25)(T), (9, 0.5)(S,M,B)	(7, 0.25)(T), (9, 0.5)(S,M)
Mixup prob.	0.8	0.8	0.8
Cutmix prob.	1.0	1.0	1.0

## Limitations of Mamba-based Networks for Visual Recognition

Although Mamba-based networks have shown state-of-the-art performance for image classification (Li et al., 2024; Zhu et al., 2024) and action recognition (Li et al., 2024), their training is unstable, which limits the scalability of these architectures. For instance, VideoMamba (Li et al., 2024) uses a distillation technique to improve training stability and performance. Since the proposed self-distillation technique requires training a smaller model first, it is a cumbersome approach that increases the training cost.

Before we propose our solution to the scalability problem in Section 4, we analyze the behavior of pure Mamba-based visual architectures in more detail. We focus on VideoMamba (Li et al., 2024) since it is the largest architecture and the only one that can be applied to video data. VideoMamba trains its tiny and small models with 7M and 25M parameters, respectively, in a conventional setting. However, distillation is used to train it as soon as the parameters are scaled up to the middle model (75M parameters) and base model (98M parameters). The method uses the smaller model as the teacher for the larger middle and base models. This is a departure from the general knowledge distillation where a larger complex model is distilled into a smaller student model (Gou et al., 2020). This reversal suggests that the purpose of distillation is not merely to transfer knowledge from a simpler model to a complex one but to stabilize the learning process of the middle and base models. As shown in Fig 1, the architecture cannot be scaled beyond 25M parameters without distillation, i.e., the accuracy does not increase further. While distillation improves the accuracy, it does not address the scaling issue since the base model is not better than the middle model. To better understand the impact of distillation on the training, we trained VideoMamba’s middle variant with and without distillation. The training curves shown in Figure 3 indicate the presence of instabilities without distillation. We also present, in Figure 3, the loss curve for our StableMamba, which has a stable convergence without distillation. To further validate our findings regarding training instabilities, we conducted additional experiments using GroupMamba (Shaker et al., 2025), a recently published architecture that exhibits similar stability issues through a different manifestation. Shaker et al. (2025) demonstrated that training instabilities manifest as high variance in loss trajectories. Without distillation regularization, training runs exhibit substantial variance and slower convergence, while incorporating a distillation loss significantly reduces variance and accelerates convergence, as shown in Fig 3.

Furthermore, we implemented our proposed stabilization method on GroupMamba, denoted by StableGroupMamba. Our approach achieves a stability comparable to that obtained through distillation regularization, providing convergent evidence for the effectiveness of our method. This additional evidence reinforces our assertion that distillation becomes necessary to mitigate or resolve these training instabilities and improve convergence, and that our proposed method can be used to address this issue without distillation.

**Fig. 7:**
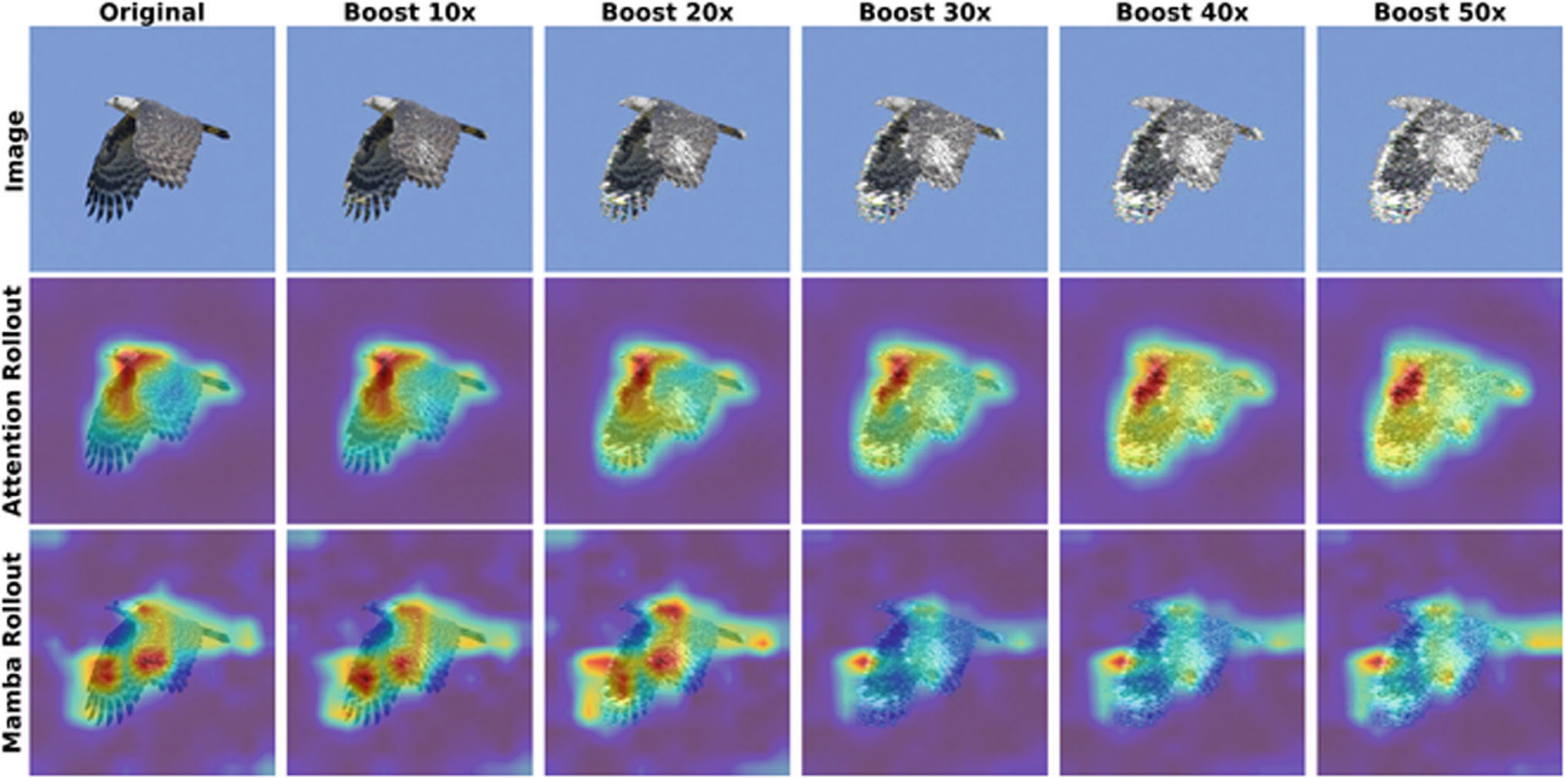
Attention rollout (Abnar & Zuidema, 2020) and Mamba rollout (Ali et al., 2025) under progressive high-frequency perturbations. Top: Input image with increasing high-frequency content. Middle: DeiT attention rollout remains consistently localized on the bird’s head across all perturbation levels. Bottom: Mamba rollout exhibits progressively degraded localization as high-frequency content increases, indicating reduced robustness to high-frequency perturbations

Furthermore, in Figure 2, we compare the behavior of VideoMamba (Li et al., 2024) with ViT-B$\backslash$16 (Dosovitskiy et al., 2021) under an increasing amount of Gaussian blurring in the input image during inference. For this, we use the images from the ImageNet-C (Hendrycks & Dietterich, 2019) benchmark, which evaluates the robustness of networks to common corruptions like Gaussian blur. As shown in Figure 2(a), VideoMamba (Li et al., 2024) suffers more than the vision Transformer from high intensities of Gaussian blurring. The better robustness of ViT-B$\backslash$16 can be explained by the fact that Transformers tend to focus on lower frequencies in the input image (Naseer et al., 2021; Park & Kim, 2022). This observation is further supported by another experiment that examines the behavior of networks under JPEG compression corruption. JPEG compression primarily removes high frequencies as the compression rate increases, although it also introduces tertiary compression-related artifacts as well. The removal of higher frequencies remains the dominant effect. Fig 2(b) shows that the VideoMamba is less robust to corruptions of higher frequencies, and addressing this challenge is an important contribution of this paper.

To further quantify the sensitivity of Mamba-based architectures to high-frequency components, we conduct a systematic frequency amplification analysis. Specifically, we transform the input images into the frequency domain using a Fast Fourier Transform (FFT) and isolate 20% of the highest frequency coefficients. These components are then scaled by a multiplicative factor to amplify their presence. After performing the inverse FFT, we obtain modified images with enhanced high-frequency content while preserving the overall content of the image. To provide a qualitative reference for the magnitude of these spectral corruptions, Figure 4 illustrates the resulting visual artifacts for a representative sample across amplification multipliers of 0$\times$ (original image), 10$\times$, and 30$\times$. We then evaluate the impact of this amplification by computing the cosine similarity between the latent tokens produced by the original image and those produced by its frequency-amplified counterpart at various multiplicative factors and various depths of the network. A low cosine similarity indicates that the internal representation of a network is significantly altered during the forward pass through the network.

As illustrated in Figure 5, the Mamba architecture demonstrates a consistently lower cosine similarity compared to the Vision Transformer across layers 1, 7, and 10. While using a multiplier of 10 does not have a noticeable impact on the internal representation of the ViT-B$\backslash$16, it strongly changes the representation of VideoMamba. We hypothesize that having large differences in the forward pass for identical images that only differ in the amplitude of high frequencies, as it can be caused by using different image scaling and compression settings, results in the high variation of the training loss of Mamba models as shown in Fig 3(b). In Section 4.3, we will discuss the theoretical perspective of this sensitivity and empirical findings.

The above-mentioned observations provide enough evidence that it is difficult to scale Mamba models. Using distillation with a smaller model is a workaround to address training instabilities for larger models since it penalizes the larger model for deviating from the smaller one and thus acts as a regularization constraint, but it does not resolve the scalability issue. Furthermore, they are less robust to common image corruptions than vision Transformers. We thus propose an efficient distillation-free solution that mitigates the scalability issue, including training stability issues for large models, and improves the robustness to common image corruptions. Our solution is motivated by the fact that vision Transformers suffer less from these issues, and we hypothesize that adding attention blocks to pure Mamba-based visual architectures resolves these issues. We evaluate the effectiveness of this hypothesis in the subsequent sections.

**Table 2:** Performance comparison on ImageNet-1K: We report the performance of our proposed models with state-of-the-art Mamba-based models and popular convolution-based and Transformer-based models on the ImageNet-1K (Deng et al., 2009) validation set. Our proposed models outperform the Mamba-based models.$^\dagger$ represents the results using distillation. ‘iso.’ means isotropic

Type	Model	iso.	Image Size	#Params (M)	FLOPs (G)	IN1K Top-1%
***CNN***	ConvNeXt-T (Liu et al., 2022a)	✗	224$^2$	29	4.5	82.1
	ConvNeXt-S (Liu et al., 2022a)	✗	224$^2$	50	8.7	83.1
	ConvNeXt-B (Liu et al., 2022a)	✗	224$^2$	89	15.4	83.8
***CNN+ SSM.***	VMamba-T (Liu et al., 2024)	✗	224$^2$	31	4.9	82.2
	VMamba-S (Liu et al., 2024)	✗	224$^2$	50	8.7	83.5
	VMamba-B (Liu et al., 2024)	✗	224$^2$	89	15.4	83.7
***Trans.***	Swin-T (Liu et al., 2021)	✗	224$^2$	28	4.6	81.3
	Swin-S (Liu et al., 2021)	✗	224$^2$	50	8.7	83.0
	Swin-B (Liu et al., 2021)	✗	224$^2$	88	15.4	83.5
	DeiT-T (Touvron et al., 2021)	✓	224$^2$	6	1.3	72.2
	DeiT-S (Touvron et al., 2021)	✓	224$^2$	22	4.6	79.8
	DeiT-B (Touvron et al., 2021)	✓	224$^2$	87	17.6	81.8
***SSM***	ViM-T (Zhu et al., 2024)	✓	224$^2$	7	1.1	76.1
	ViM-S (Zhu et al., 2024)	✓	224$^2$	26	4.3	80.5
	VideoMamba-T (Liu et al., 2024)	✓	224$^2$	7	1.1	76.9
	VideoMamba-S (Liu et al., 2024)	✓	224$^2$	26	4.3	81.2
	VideoMamba-M (Liu et al., 2024)	✓	224$^2$	74	12.7	81.4
	VideoMamba-$\hbox {M}^\dagger$ (Liu et al., 2024)	✓	224$^2$	74	12.7	82.8
	VideoMamba-$\hbox {B}^\dagger$ (Liu et al., 2024)	✓	224$^2$	98	16.9	82.7
	StableMamba-T	✓	224$^2$	7	1.2	77.4
	StableMamba-S	✓	224$^2$	27	4.4	81.5
	StableMamba-M	✓	224$^2$	76	12.9	83.1
	StableMamba-$\hbox {M}^\dagger$	✓	224$^2$	76	12.9	83.5
	StableMamba-B	✓	224$^2$	101	17.1	83.9
	StableMamba-$\hbox {B}^\dagger$	✓	224$^2$	101	17.1	84.1
	StableMamba-L	✓	224$^2$	187	33.7	84.6
	GroupMamba-S	✓	224$^2$	39.2	8.1	83.2
	GroupMamba-$\hbox {S}^\dagger$	✓	224$^2$	39.2	8.1	84.0
	StableGroupMamba-S	✓	224$^2$	39.3	8.3	83.8
	StableGroupMamba-$\hbox {S}^\dagger$	✓	224$^2$	39.3	8.3	84.2

**Table 3:** Comparison with state-of-the-art methods on Kinetics-400 (Kay et al., 2017). $^\dagger$ represents initialization with ImageNet-1K pretraining using distillation

Arch.	Model	P.T.	Input Size	#Params (M)	FLOPs (G)	K400 Top-1%
***CNN***	$\hbox {SlowFast}_{R101+NL}$	-	80$\times$224$^2$	60	234$\times$3$\times$10	79.8
	(Feichtenhofer et al., 2019)					
	X3D-M (Feichtenhofer, 2020)	-	16$\times$224$^2$	4	6$\times$3$\times$10	76.0
	X3D-XL (Feichtenhofer, 2020)	-	16$\times$312$^2$	20	194$\times$3$\times$10	80.4
***CNN+ Trans.***	MViTv1-B (Fan et al., 2021)	–	32$\times$224$^2$	37	70$\times$1$\times$5	80.2
	MViTv2-S (Li et al., 2022a)	-	16$\times$224$^2$	35	64$\times$1$\times$5	81.0
	UniFormer-S (Li et al., 2022b)	IN1K	16$\times$224$^2$	21	42$\times$1$\times$4	80.8
	UniFormer-B (Li et al., 2022b)	IN1K	16$\times$224$^2$	50	97$\times$1$\times$4	82.0
	UniFormer-B (Li et al., 2022b)	IN1K	32$\times$224$^2$	50	259$\times$3$\times$4	83.0
***Trans.***	Swin-T (Liu et al., 2022b)	IN1K	32$\times$224$^2$	28	88$\times$3$\times$4	78.8
	Swin-B (Liu et al., 2022b)	IN1K	32$\times$224$^2$	88	88$\times$3$\times$4	80.6
	Swin-B (Liu et al., 2022b)	IN21K	32$\times$224$^2$	88	282$\times$3$\times$4	82.7
	STAM (Sharir et al. 2021)	IN21K	64$\times$224$^2$	121	1040$\times$1$\times$1	79.2
	TimeSformer-L	IN21K	96$\times$224$^2$	121	2380$\times$3$\times$1	80.7
	(Bertasius et al. 2021)					
	ViViT-L (Arnab et al., 2021)	IN21K	16$\times$224$^2$	311	3992$\times$3$\times$4	81.3
	Mformer-HR (Patrick et al., 2021)	IN21K	16$\times$336$^2$	311	959$\times$3$\times$10	81.1
***SSM***	VideoMamba-T (Li et al., 2024)	IN1K	16$\times$224$^2$	7	17$\times$3$\times$4	78.1
	VideoMamba-S (Li et al., 2024)	IN1K	16$\times$224$^2$	26	68$\times$3$\times$4	80.8
	VideoMamba-$\hbox {M}^\dagger$ (Li et al., 2024)	IN1K	16$\times$224$^2$	74	202$\times$3$\times$4	81.9
	StableMamba-T	IN1K	16$\times$224$^2$	7	19$\times$3$\times$4	78.6
	StableMamba-S	IN1K	16$\times$224$^2$	27	70$\times$3$\times$4	81.2
	StableMamba-M	IN1K	16$\times$224$^2$	76	206$\times$3$\times$4	82.2
	StableMamba-$\hbox {M}^\dagger$	IN1K	16$\times$224$^2$	76	206$\times$3$\times$4	82.5
	StableMamba-B	IN1K	16$\times$224$^2$	101	303$\times$3$\times$4	82.8

## StableMamba for Image Classification and Action Recognition

Before discussing the StableMamba architecture in Section 4.2, we briefly introduce state-space models in general.

### State-Space Models

State-space models (SSMs) are inspired by continuous systems in which an input signal *u*(*t*) is mapped to a latent state *h*(*t*) before being mapped to an output signal *y*(*t*). Concretely, a linear ordinary differential equation describes the SSM model:

1 $$\begin{aligned} \begin{aligned} h'(t)&= {{\textbf{A}}}h(t) + {{\textbf{B}}}u(t) \\ y(t)&= {{\textbf{C}}}h(t) \end{aligned} \end{aligned}$$

where *h*(*t*) is the hidden state, $h'(t)$ is the first derivative, *u*(*t*) is the input, and *y*(*t*) is the output. $\textbf{A}$ is the evolution matrix, and $\textbf{B}$ and $\textbf{C}$ are the projection matrices of the system.

**Discretization of State-Space Models:** As mentioned before, Equation (1) is valid for continuous time systems. To apply Equation (1) on a discretized input sequence $(u_0, u_1, u_2,...)$ instead of a continuous function *u*(*t*), Equation (1) must be discretized using a step size $\Delta$ which describes the input time-step resolution. The standard discretization that follows Mamba (Gu & Dao, 2024) is the Zero-Order Hold (ZOH) discretization:

2 $$\begin{aligned} \begin{aligned} \overline{{{\textbf{A}}}}&= \exp (\Delta {\textbf{A}}) \\ \overline{{{\textbf{B}}}}&= (\Delta {\textbf{A}})^{-1} (\exp (\Delta {\textbf{A}}) - {{\textbf{I}}}) \cdot \Delta {\textbf{B}} \\ h_t&= \overline{{{\textbf{A}}}} h_{t-1} + \overline{{{\textbf{B}}}} u_t \\ y_t&= {{\textbf{C}}}h_t. \end{aligned} \end{aligned}$$

The difference between S4 (Gu et al., 2022) and Mamba (Gu & Dao, 2024) is the selective scan mechanism that conditions the parameters of ${\textbf{A}}$, ${\textbf{B}}$, and ${\textbf{C}}$ on input.

**Table 4:** Comparison with state-of-the-art methods on the Something-Something-v2 (Goyal et al., 2017) dataset. $^\dagger$ represents initialization with ImageNet-1K pretraining using distillation. Network input sizes are the same as mentioned in K400

Arch.	Model	P.T.	#Params (M)	FLOPs (G)	**SSv2 Top-1%**
***CNN***	$\hbox {SlowFast}_{R101}$	K400	53	106$\times$3$\times$1	63.1
	(Feichtenhofer et al., 2019)				
	CT-$\hbox {Net}_{R50}$ (Li et al., 2020)	IN1K	21	75$\times$1$\times$1	64.5
	$\hbox {TDN}_{R50}$ (Wang et al., 2021)	IN1K	26	75$\times$1$\times$1	65.3
***CNN+ Trans.***	MViTv1-B (Fan et al., 2021)	K400	37	71$\times$3$\times$1	64.7
	MViTv1-B (Fan et al., 2021)	K400	37	170$\times$3$\times$1	67.1
	MViTv2-S (Li et al., 2022a)	K400	35	65$\times$3$\times$1	68.2
	MViTv2-B (Li et al., 2022a)	K400	51	225$\times$3$\times$1	70.5
	UniFormer-S (Li et al., 2022b)	IN1K+K400	21	42$\times$3$\times$1	67.7
	UniFormer-B (Li et al., 2022b)	IN1K+K400	50	97$\times$3$\times$1	70.4
***Trans.***	Swin-B (Liu et al., 2022b)	K400	89	88$\times$3$\times$1	69.6
	ViViT-L (Arnab et al., 2021)	IN21K+K400	311	3992$\times$3$\times$4	65.4
	Mformer-HR (Patrick et al., 2021)	IN21K+K400	311	1185$\times$3$\times$1	68.1
	TimeSformer-HR	IN21K	121	1703$\times$3$\times$1	62.5
	(Bertasius et al. 2021)				
***SSM***	VideoMamba-T (Li et al., 2024)	IN1K	7	9$\times$3$\times$2	65.1
	VideoMamba-S (Li et al., 2024)	IN1K	26	34$\times$3$\times$2	66.6
	VideoMamba-$\hbox {M}^\dagger$ (Li et al., 2024)	IN1K	74	101$\times$3$\times$4	67.3
	StableMamba-T	IN1K	7	10$\times$3$\times$2	65.7
	StableMamba-S	IN1K	27	35$\times$3$\times$2	67.3
	StableMamba-M	IN1K	76	103$\times$3$\times$4	67.8
	StableMamba-$\hbox {M}^\dagger$	IN1K	76	103$\times$3$\times$4	68.1

### StableMamba

**Table 5:** Mean Corruption Error (mCE) on the ImageNet-C (Hendrycks & Dietterich, 2019) dataset across all 19 corruptions. mCE is reported relative to AlexNet (Krizhevsky et al., 2012) errors on ImageNet-C

Model	Error on Clean	Mean Corruption Error (mCE)
AlexNet	43.48%	100.0%
SqueezeNet1.1	41.82%	104.4%
VGG11	30.98%	93.5%
VGG19	27.62%	88.9%
VGG19BN	25.78%	81.6%
DenseNet121	25.57%	73.4%
DenseNet169	24.40%	69.4%
DenseNet201	23.10%	68.4%
DenseNet161	22.86%	66.4%
CondenseNet4	26.25%	80.8%
CondenseNet8	28.93%	84.6%
ResNet18	30.24%	84.7%
ResNet34	26.69%	77.9%
ResNet50	23.87%	76.7%
ResNet101	22.63%	70.4%
ResNet152	21.69%	69.3%
ResNeXt50	22.89%	68.2%
ResNeXt101	21.81%	63.6%
ResNeXt101_64	21.04%	62.2%
ViT-B/16	22.10%	53.7%
DeiT-B	18.20%	50.4%
VideoMamba-M	18.60%	51.6%
StableMamba-M	16.90%	50.5%

VideoMamba (Li et al., 2024) uses bi-directional Mamba layers introduced by VisionMamba (Zhu et al., 2024) and shown in Fig 6(d). A bi-directional Mamba block adapts the concept of bi-directional sequence modeling to vision-related tasks. It processes flattened visual token sequences simultaneously using forward and backward state-space models.

Our architecture consists of stacked StableMamba blocks. Within each StableMamba block are *N* bi-directional Mamba blocks and *A* Transformer blocks as shown in Fig 6(a). The purpose of the Transformer blocks is to stabilize the training and increase the robustness by resetting the focus after several bi-directional Mamba blocks more on lower frequencies. We will evaluate the impact of the number of Transformer blocks in each StableMamba block and the position of the Transformer block within the StableMamba block in Section 5. We now describe the two blocks in more detail.

**Transformer block:** The Transformer block is detailed in Figure 6(b). Each Transformer block begins with a Root Mean Square (RMS) normalization layer applied to the input data. It follows a Self-Attention layer where three learnable linear layers $\textbf{W}^Q$, $\textbf{W}^K$, and $\textbf{W}^V$ are used for transforming the input $\textbf{X}$ into queries ($\textbf{Q}$), keys ($\textbf{K}$) and values ($\textbf{V}$) such that $\textbf{Q}=\textbf{X}\textbf{W}^Q$, $\textbf{K}=\textbf{X}\textbf{W}^K$, and $\textbf{V}=\textbf{X}\textbf{W}^V$. The output $\textbf{Z}$ of the Self-Attention layer is then calculated as:

3 $$\begin{aligned} \textbf{Z}= \textsf {SOFTMAX}\left( \frac{\textbf{Q}\textbf{K}^T}{\sqrt{D_q}}\right) \textbf{V} \end{aligned}$$

where $D_q$ is the dimension of the query; furthermore, a skip connection is added to the output. Subsequently, another RMS normalization is applied, after which this output is fed to an MLP layer. This constitutes the entire Transformer block shown in Fig 6(b). The operations can be summarized as:

4 $$\begin{aligned} \begin{aligned} \textbf{Z}_{\textrm{in}}&= \textsf {PE} + \textsf {EMB}(\textbf{X}) \\ \textbf{Z}'_{\textrm{out}}&= \textbf{Z}_{\textrm{in}} + \textsf {ATTN}(\textsf {RMSNORM}(\textbf{Z}_{\textrm{in}})) \\ \textbf{Z}_{\textrm{out}}&= \textbf{Z}'_{\textrm{out}} + \textsf {MLP}(\textsf {RMSNORM}(\textbf{Z}'_{\textrm{out}})) \end{aligned} \end{aligned}$$

where $\textbf{X}$ is the input to the Transformer block. $\textsf {EMB}$ is the convolutional patch embedding and $\textsf {PE}$ is the positional encoding as in (Dosovitskiy et al., 2021). $\textsf {RMSNORM}$ is the RMS norm layer and $\textsf {ATTN}$ denotes the multi-head Self-Attention layer described in Equation (3). The $\textsf {MLP}$ is defined by:

5 $$\begin{aligned}&\textsf {MLP}(\textsf {RMSNORM}(\textbf{Z}'_{\textrm{out}})) =\nonumber \\&\quad \textsf {GELU}(\textsf {RMSNORM}(\textbf{Z}'_{\textrm{out}}) \textbf{W}_1 + \textbf{b}_1) \times \textbf{W}_2 + \textbf{b}_2. \end{aligned}$$

**Fig. 8:**
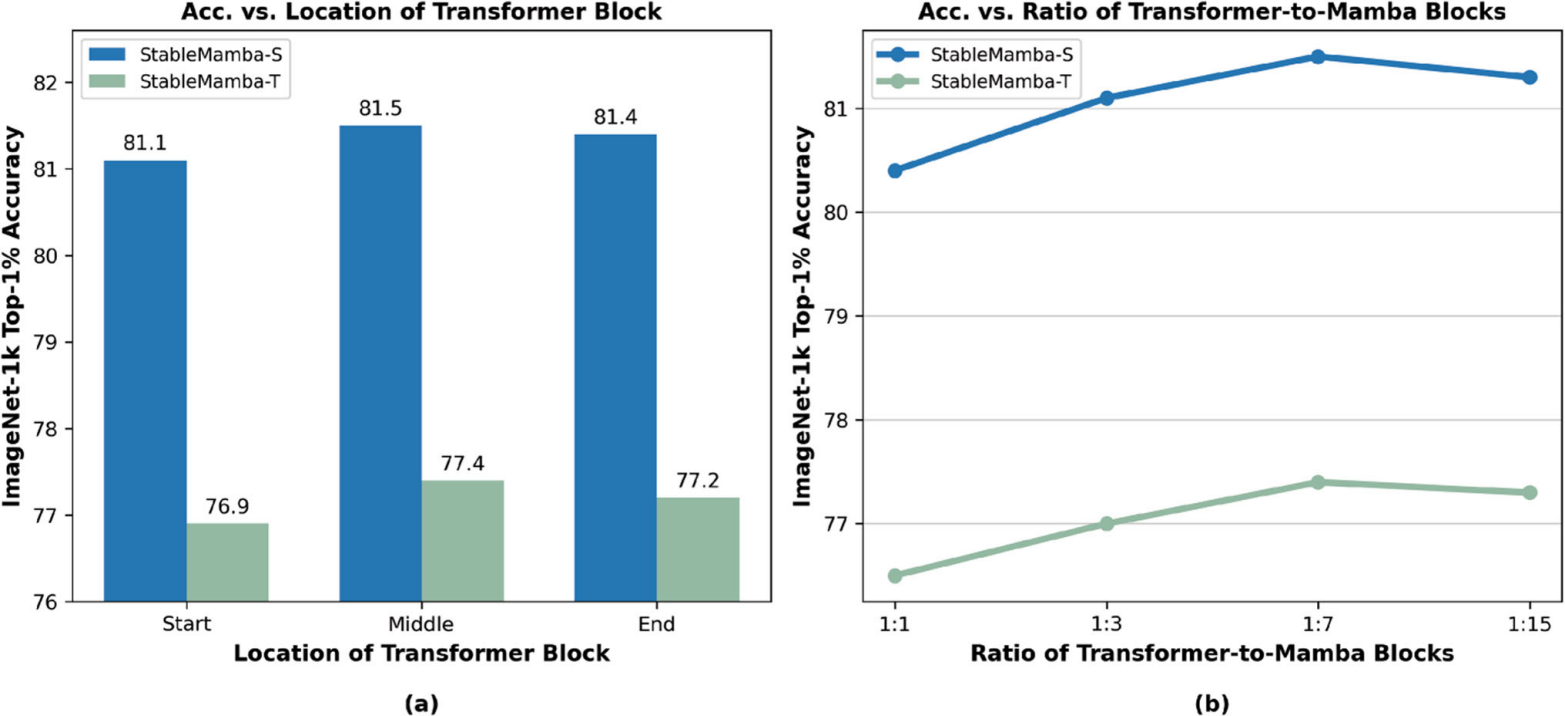
**(a)** Impact of the position of the Transformer block within StableMamba. **(b)** Impact of the ratio of Transformer blocks to Mamba blocks

**Mamba block:** The Mamba block (Fig 6(c)) has the same structure as the Transformer block except that it uses a bi-directional Mamba layer instead of a self-attention layer. For brevity’s sake, we will call the bi-directional Mamba layer simply as the Mamba layer. The Mamba block performs the following operations:

6 $$\begin{aligned} \begin{aligned} \textbf{Z}'_{\textrm{out}}&= \textbf{Z}_{\textrm{in}} + \textsf {MAMBA}(\textsf {RMSNORM}(\textbf{Z}_{\textrm{in}})) \\ \textbf{Z}_{\textrm{out}}&= \textbf{Z}'_{\textrm{out}} + \textsf {FFN}(\textsf {RMSNORM}(\textbf{Z}'_{\textrm{out}})). \end{aligned} \end{aligned}$$

Our Mamba block differs from VideoMamba (Li et al., 2024) in that we add an RMS normalization layer and an MLP layer inside the Mamba block.

The number of parameters of the network can be controlled by the depth of the network and the embedding dimension. We introduce four variations of our model: StableMamba-Tiny has 7M parameters, StableMamba-Small has 27M parameters, StableMamba-Middle has 76M parameters, StableMamba-Base has 101M parameters, and StableMamba-Large has 187M parameters. The complete list of hyperparameters for reproducibility purposes is provided in Table 1. We use 4 nodes with 4 A100 GPUs (40GB) each for training all of our StableMamba models.

### Theoretical Perspective for Mamba’s Sensitivity

The sensitivity of Mamba to high frequency perturbations, as shown in Section 3, can be understood through the structural differences between selective SSMs and attention blocks. Han et al. (2024) showed that the selective SSM from Equation (1) can be rewritten (7) in a formulation comparable to linear attention (8):

7 $$\begin{aligned} h_t&= \tilde{A}_t \odot h_{t-1} + {{\textbf{B}}}_t(\Delta _t \odot u_t),&y_t&= {{\textbf{C}}}_t h_t \;/\; 1 \end{aligned}$$

8 $$\begin{aligned} S_t&= 1 \odot S_{t-1} + K_t^\top (1 \odot V_t),&y_t&= Q_t S_t \;/\; Q_t Z_t \end{aligned}$$

In Equation (7), *t* indexes the sequence position (time step), $u_t$ denotes the input at step *t*, $h_t$ is the selective SSM hidden state, and $y_t$ is the corresponding output. The operator $\odot$ denotes the Hadamard product, and 1 denotes all-ones vectors (or tensors) of appropriate shape. Since the matrix $\overline{{{\textbf{A}}}}_t$ is in practice a diagonal matrix, $\tilde{A}_t$ is the vector of the elements of $\overline{{\textbf{A}}}_t$ on the diagonal. The quantity $\tilde{A}_t$ is an input-dependent forget or decay gate applied to the previous state $h_{t-1}$, $\Delta _t$ is an (input-dependent) input gate applied to $u_t$, and $B_t$ and $C_t$ are (input-dependent) linear maps that project the gated input into the state space and the state into the output space, respectively. In Equation (8), $S_t$ denotes the (linear attention) running state or accumulator, $Q_t$ is the query at step *t*, $K_t$ and $V_t$ are the key and value at step *t*. The term $Z_t$ denotes the (linear attention) normalizer accumulator, which corresponds often to an accumulated feature map of keys, and the denominator $Q_t Z_t$ provides an additional normalization.

As discussed in Han et al. (2024), a side-by-side comparison of Equation (7) and Equation (8) allows an interpretation of Mamba as a variant of single-head linear attention augmented with an input-dependent forget gate $\tilde{A}_t$ and an input gate $\Delta _t$ but without attention normalization. Han et al. (2024) have demonstrated empirically that removing the normalization by $Q_t Z_t$ in Equation (8) causes the standard deviation of token norms to grow dramatically across layers, allowing higher-magnitude tokens to dominate the feature map while suppressing others. When high frequencies are amplified as in Section 3, the magnitudes of certain tokens become disproportionately large. Without normalization they overwhelm the hidden state $h_t$ in Mamba’s recurrence, whereas softmax or linear attention’s denominator $Q_t Z_t$ re-normalizes and attenuates this effect. This leads to training instabilities and high sensitivity to high-frequency perturbations as shown in Figure 7 using attention rollout (Abnar & Zuidema, 2020) for transformer and Mamba rollout (Ali et al., 2025) for the Mamba-based network. While DeiT’s attention rollout retains meaningful even if high frequencies are strongly amplified, Mamba’s rollout attribution maps degrade progressively with increasing high-frequency amplification, eventually becoming nearly random. Honarpisheh et al. (2025) also compare the generalization bounds of specific variants of selective SSMs and linear attention models. While the bound of linear attention scales with $\mathfrak B_u^3$, where $\mathfrak B_u$ is the upper bound of the input’s $l_2$-norm, i.e., $\Vert u_t\Vert _2\le \mathfrak B_u$ for all *t*, the bound of the selective SSMs scales as $\mathfrak B_u^4$. This theoretical analysis also explains why selective SSMs are inherently more sensitive to input magnitudes than attention-based models.

## Results

We evaluate our model for image classification on ImageNet-1K (IN1K) (Deng et al., 2009) and for video recognition on Kinetics-400 (K400) (Kay et al., 2017) and Something-Something-v2 (SSv2) (Goyal et al., 2017). For evaluating the robustness to various common corruptions, we use the ImageNet-C (IN-C) (Hendrycks & Dietterich, 2019) benchmark. Note that ImageNet-C is only used for testing, but not for training.

**Table 6:** Impact of image resolution (top) and number of input frames (bottom) for StableMamba and VideoMamba

Model	Context Length	Training Dataset	FLOPs (G)	Accuracy
VideoMamba-T	$224^2$	IN1K	1.1	76.9%
StableMamba-T	$224^2$	IN1K	1.2	**77.4%**
VideoMamba-T	$448^2$	IN1K	4.3	79.3%
StableMamba-T	$448^2$	IN1K	4.5	**79.9%**
VideoMamba-T	$16\times 224^2$	K400	$17\times 3\times 4$	78.1%
StableMamba-T	$16\times 224^2$	K400	$19\times 3\times 4$	**78.6%**
VideoMamba-T	$32\times 224^2$	K400	$34\times 3\times 4$	78.8%
StableMamba-T	$32\times 224^2$	K400	$37\times 3\times 4$	**79.3%**

### Evaluation on ImageNet-1K

We use the IN1K (Deng et al., 2009) dataset for pre-training our models. IN1K contains 1.28M training and 50k validation images for 1000 categories. The models pre-trained on IN1K are used as an initializing point for fine-tuning on the other datasets.

**Evaluation Setup:** We train our models for 300 epochs on IN1K, using the AdamW optimizer (Loshchilov & Hutter, 2017) with a learning rate of 5e-4, weight decay of 0.1 for the tiny model and 0.05 for the other models, a batch size of 128 per GPU, input image resolution of 224, and a patch size of 16. We set the ratio of Transformer blocks to Mamba blocks to 1:7 for our baseline models. We use 4 nodes with 4 A100 GPUs (40GB) each for training. We do not use any automatic mixed precision. For a fair comparison, we also train our models with and without distillation to gauge the effect of distillation on the overall training scheme and architecture. Following VideoMamba (Li et al., 2024), use the spatial-first bidirectional scan for images. The complete set of hyperparameters is provided in Table 1.

To provide a comprehensive evaluation, we conducted additional experiments comparing our approach with the GroupMamba architecture (Shaker et al., 2025). The implementation required modifying the third-stage embedding dimension from 348 to 384 to ensure compatibility with multi-head attention mechanisms. This architectural adjustment increased the parameter count to 39M for both distilled and non-distilled variants. We applied our method to GroupMamba by systematically replacing alternating Visual Single Selective Scanning (VSSS) blocks with attention blocks while preserving the original interleaving pattern and ratio, creating StableGroupMamba. The resulting architecture exhibits a modest parameter increase (39.3M vs. 39.2M) compared to the baseline due to the computational requirements of multi-head attention exceeding those of 2D selective scan operations.

Note that the distillation strategies employed by GroupMamba and VideoMamba differ substantially in their teacher model selection. GroupMamba utilizes a significantly larger RegNet-Y architecture as the teacher model, whereas VideoMamba employs smaller models from within their own architectural family. This fundamental difference in teacher model capacity accounts for the more pronounced performance improvements observed in GroupMamba experiments, as larger teacher models typically provide richer supervisory signals during knowledge distillation.

**Results:** We present results for evaluating StableMamba on the IN1K dataset with other comparable methods in Table 2. We train our method with and without distillation to show the impact of distillation on the accuracy. We first compare the results without distillation. StableMamba outperforms the current state-of-the-art isotropic visual SSM models (ViM and VideoMamba) on IN1K for all model sizes. Compared to VideoMamba, the improvement ($+1.7$) of StableMamba is largest for the model M, which is the largest model of VideoMamba that can be trained without distillation. Note that an improvement of $+1.7$ on IN1K is substantial. The improvements compared to VideoMamba are visualized by the solid lines in Figure 1, which show the lack of scalability of VideoMamba. If we compare VideoMamba and StableMamba with distillation, we observe that distillation improves the accuracy for both architectures, but StableMamba still outperforms VideoMamba. The accuracy of StableMamba-$\hbox {B}^\dagger$ is $+1.4$ higher than that of VideoMamba-$\hbox {B}^\dagger$. It is interesting to note that StableMamba-B without distillation even outperforms VideoMamba-$\hbox {B}^\dagger$ with distillation by $+1.2$. The trend continues with StableMamba-L having 84.6% top-1 accuracy against 83.9% of StableMamba-B, i.e., an improvement of +0.7. Most important, however, is that StableMamba can be scaled up and does not need any distillation as shown in Figure 1.

For further evidence of the effectiveness of our technique, we train GroupMamba-S (Shaker et al., 2025) with the attention interleaved layers, denoted by StableGroupMamba-S. The results in Table 2 show that it has better performance than GroupMamba-S without distillation and converges better as shown in Fig3b.

### Evaluation on Video Recognition

**Fig. 9:**
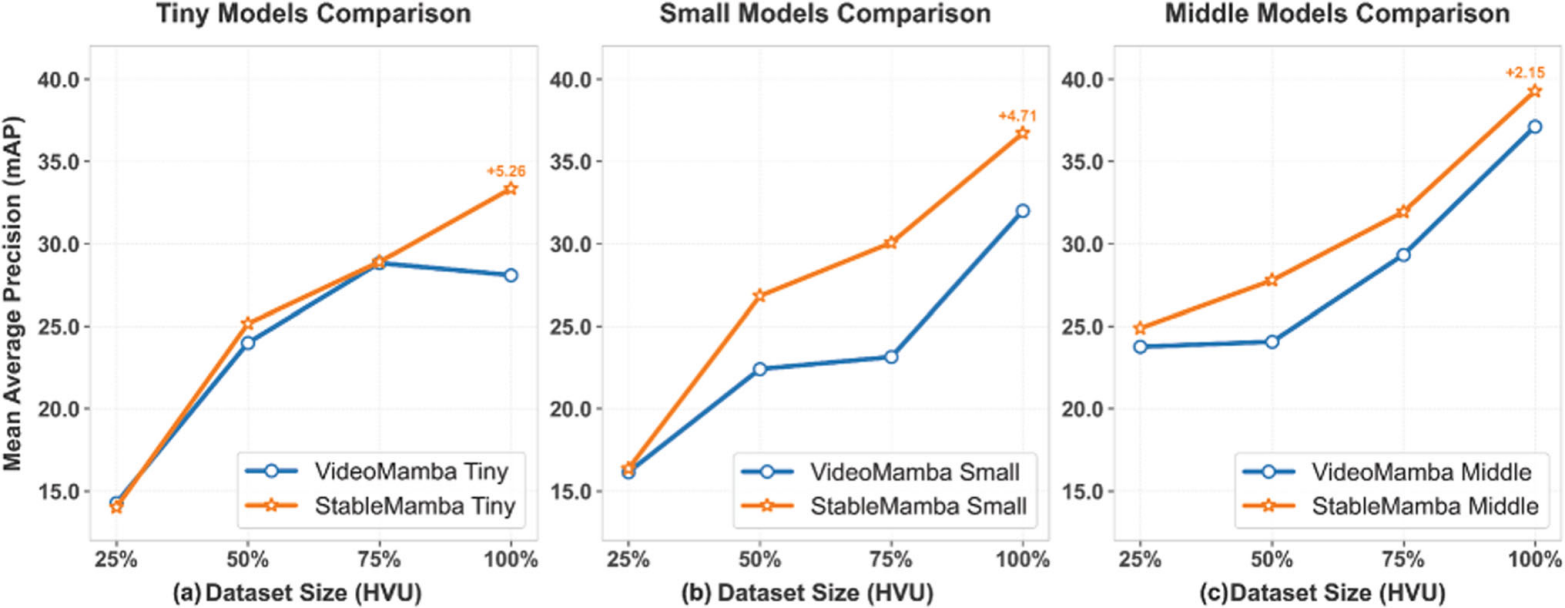
**(a)** Dataset scaling experiment using 25%, 50%, 75%, and 100% of the training dataset while performing the validation on the full validation set

After pre-training on IN1K, we fine-tune the models on two large-scale datasets. The first dataset, K400 (Kay et al., 2017), includes approximately 240,000 training videos and 19,000 validation videos, each about 10 seconds long, spanning 400 different human action classes. The second dataset, SSv2 (Goyal et al., 2017), consists of around 220,000 videos: 168,000 for training, 24,000 for validation, and 27,000 for testing, covering 174 different classes.

**Evaluation Setup:** For fine-tuning, we use a batch size of 32 for tiny and a batch size of 16 for the other variants due to the GPU memory limit. We set the number of linear warm-up epochs to 5, and the total number of epochs to 70 for K400 and 35 for SSv2 as in (Li et al., 2024) for the tiny model, and 50 and 30, respectively, for the other models. We use AdamW as an optimizer. We use the same spatiotemporal scan as used by VideoMamba (Li et al., 2024). The complete list of hyperparameters for reproducibility is provided in Table 1.

**Results:** StableMamba demonstrates superior performance in downstream video recognition tasks compared to VideoMamba, which is the only Mamba architecture that can be applied to videos. On the K400 dataset in Table 3, StableMamba tiny and small outperform their VideoMamba counterparts without distillation. Distillation improves the accuracy for the middle models, but even with distillation, StableMamba-$\hbox {M}^\dagger$ improves the accuracy of VideoMamba-$\hbox {M}^\dagger$ by $+0.6$, which is a substantial improvement on this dataset. StableMamba-B extends this trend further, achieving $+0.6$ improvement over StableMamba-M and $+0.3$ improvement over StableMamba-$\hbox {M}^\dagger$, establishing new state-of-the-art performance on this benchmark. The results on the SSv2 dataset shown in Table 4 are similar, but the improvements are even larger. StableMamba-$\hbox {M}^\dagger$ improves on the accuracy of VideoMamba-$\hbox {M}^\dagger$ by $+0.8$.

**Fig. 10:**
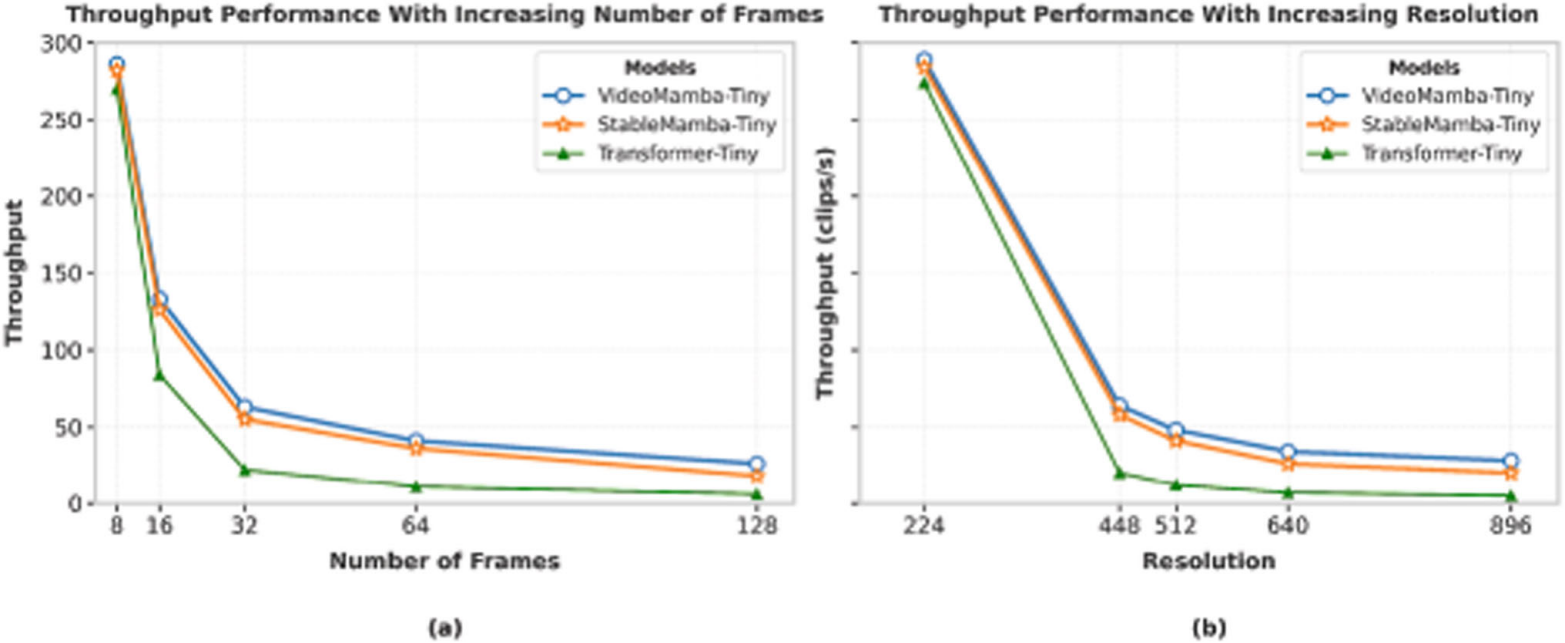
Throughput plot for VideoMamba and StableMamba. Even with attention blocks, StableMamba remains competitive on all temporal length sequences with a pure Mamba-based model. The Transformer-Tiny model is made by replacing all the Mamba blocks in the StableMamba-Tiny model with Transformer blocks

### Evaluation on ImageNet-C

IN-C (Hendrycks & Dietterich, 2019) is a benchmark for evaluating the robustness of neural networks to images with common corruptions like JPEG compression. It includes 19 common types of image corruption at 5 different intensity levels. We test our network on this benchmark to assess the robustness introduced by attention layers.

**Results:** We present results for Gaussian blurring and JPEG compression corruption for StableMamba-M in comparison with VideoMamba-M, ViT-B$\backslash$16, and ResNet-50 in Figure 2. We see that StableMamba-M (blue) outperforms VideoMamba-M (yellow) for all levels of corruption. The gap becomes larger as the intensity of corruption increases. StableMamba behaves similarly or even slightly better than the pure attention-based architecture ViT-B$\backslash$16 and is more robust than ResNet-50, in particular for the highly relevant JPEG compression setting.

We also report the results across all corruptions in Table 5. The Mean Corruption Error (mCE) on the ImageNet-C dataset presented in Table 5 showcases the robustness of various models to common image corruptions, with errors reported relative to AlexNet. Our proposed model, StableMamba-M, demonstrates superior performance with an mCE of 50.5%, which is competitive with the DeiT-B model, which has an mCE of 50.4%. Notably, StableMamba-M outperforms ViT-B/16 and VideoMamba-M, which have mCEs of 53.7% and 51.6%, respectively, highlighting its improved robustness. This comparison underscores StableMamba’s effectiveness in enhancing model stability and corruption resistance, providing a significant advancement over existing models like VideoMamba.

### Ablation Studies

**Position of Transformer Blocks:** In Figure 6(a), the Transformer block is placed in the middle of the StableMamba blocks. This position results from our analysis of the impact on the location of the Transformer block. We conducted three experiments each for StableMamba-T and StableMamba-S, totaling six experiments, to determine the optimal position for the Transformer block. We tested placing the Transformer block at the start, middle, and end of the StableMamba blocks and evaluated their performance on the IN1K dataset. As shown in Figure 8(a), the performance of StableMamba is not highly sensitive to the Transformer’s position in both tiny and small models. However, there is a slight performance improvement when the Transformer block is in the middle. Therefore, we use the middle position as the default for our StableMamba architecture.

**Number of Transformer Blocks:** Similar to the position of Transformer blocks within each StableMamba block, the ratio of Transformer blocks to Mamba blocks is another design parameter for the StableMamba block. We interleave a Transformer block for every *k* Mamba block; for example, we interleave one Transformer block for every seven Mamba blocks. To evaluate the impact of the ratio, we conducted experiments varying the number of Mamba blocks per Transformer block. As shown in Figure 8(b), the performance on the IN1K dataset improves as the number of Mamba blocks per Transformer block increases, reaching optimal accuracy at a ratio of 1:7. Beyond this ratio, the performance decreases. Therefore, we set the design parameter to one Transformer block for every seven Mamba blocks in the StableMamba architecture.

**Impact of Context Length:** Apart from the network architecture itself, it is interesting to investigate the network with context lengths of different sizes. To probe the suitability of our approach for a long context, we perform additional experiments. First, we train StableMamba-T with a longer context for video classification, using 32 frames instead of the usual 16 frames. Second, we train StableMamba with a larger resolution (448 instead of 224) to see its effect on image classification as well. The results in Table 6 show that StableMamba and VideoMamba benefit from the increased context length, which is a general strength of Mamba-based architectures. In all cases, StableMamba outperforms VideoMamba.

**Impact of Dataset Size:** Along with the context length, it is also interesting to ablate the data efficiency of the network. For this purpose, we conducted scaling experiments using 25%, 50%, 75%, and 100% of the training dataset while performing the validation on the full validation set. The results in Figure 9 show that our network consistently outperforms the VideoMamba model across all data regimes. While conventional approaches exhibit performance saturation as data volume increases, our architecture maintains higher accuracy at each threshold and continues to improve with additional data. The performance gap is already evident at the 25% level for small and middle models and progressively widens with dataset scaling, confirming that our modifications enable better representation learning from limited samples without compromising the ability to leverage larger datasets.

**Impact on Throughput:** To evaluate the computational impact of incorporating attention mechanisms with extended temporal receptive fields, we conducted a throughput analysis of the StableMamba-T architecture. We measured processing throughput (clips per second) across varying temporal sequence lengths, systematically evaluating performance from 8-frame to 128-frame video clips. As shown in Figure 10, the integration of transformer blocks within the StableMamba architecture introduces a negligible computational overhead. The throughput characteristics only marginally degrade compared to those of the baseline VideoMamba-T model across all evaluated sequence lengths, indicating that our stabilization approach maintains computational efficiency while providing enhanced training stability.

We also evaluate the performance of our approach at the architectural extreme where all blocks are replaced with Transformer layers, effectively creating a pure Transformer model. As anticipated, this configuration substantially increases computational complexity and reduces inference throughput. Consequently, our approach maintains greater architectural and computational closeness to the original Mamba design while achieving improved stability compared to pure Mamba architectures.

## Conclusion

We have investigated and addressed the scalability challenge in large visual state-space models by proposing a straightforward interleaved design that scales effectively to a substantial number of parameters, consistently outperforming smaller models. Our ablation studies provide insights regarding optimal positioning, the number of attention layers in the architecture, and its robustness to common corruptions in the input like JPEG compression. Extensive experiments show that our method enables the scaling of Mamba-based models to over 180M parameters, significantly enhancing performance while also improving overall robustness. Evaluations on the K400 and SSv2 datasets for video recognition validate that our approach achieves state-of-the-art results.

## Data Availability

Not applicable

## Declarations

### Code availability

Code will be made publicly available on Github.
